# Retraction Note: Comparing ion transport in ionic liquids and polymerized ionic liquids

**DOI:** 10.1038/s41598-024-70285-x

**Published:** 2024-08-21

**Authors:** Wangchuan Xiao, Quan Yang, Shenlin Zhu

**Affiliations:** 1 School of Resources and Chemical Engineering, Sanming University, Fujian, 365004 China; 2 Department of Chemical Engineering, Tsinghua University, Beijing, 100084 China

Retraction of: *Scientific Reports* 10.1038/s41598-020-64689-8, published online 08 May 2020

The Editors have retracted this Article.

After publication, concerns were raised about the methodology described and the authorship. The Editors requested that the Authors provide the original data and more detail with respect to the methods used in the study. However, the response from the Authors was unsatisfactory. Additionally, the Editors have been unable to verify the authorship of this work. The Editors therefore no longer have confidence in the validity of the results and conclusions presented.

Wangchuan Xiao and Quan Yang have not responded to correspondence from the Editors about this retraction. The Editors were not able to obtain a current email address for Shenlin Zhu.

